# Fast and inexpensive protocols for consistent extraction of high quality DNA and RNA from challenging plant and fungal samples for high-throughput SNP genotyping and sequencing applications

**DOI:** 10.1371/journal.pone.0206085

**Published:** 2018-10-18

**Authors:** Peter W. Inglis, Marilia de Castro R. Pappas, Lucileide V. Resende, Dario Grattapaglia

**Affiliations:** 1 Plant Genetics Laboratory, Embrapa Genetic Resources and Biotechnology, Brasília, Brazil; 2 Genomic Sciences and Biotechnology Program, Universidade Católica de Brasília, Brasília, Brazil; University of Helsinki, FINLAND

## Abstract

Modern genotyping techniques, such as SNP analysis and genotyping by sequencing (GBS), are hampered by poor DNA quality and purity, particularly in challenging plant species, rich in secondary metabolites. We therefore investigated the utility of a pre-wash step using a buffered sorbitol solution, prior to DNA extraction using a high salt CTAB extraction protocol, in a high throughput or miniprep setting. This pre-wash appears to remove interfering metabolites, such as polyphenols and polysaccharides, from tissue macerates. We also investigated the adaptability of the sorbitol pre-wash for RNA extraction using a lithium chloride-based protocol. The method was successfully applied to a variety of tissues, including leaf, cambium and fruit of diverse plant species including annual crops, forest and fruit trees, herbarium leaf material and lyophilized fungal mycelium. We consistently obtained good yields of high purity DNA or RNA in all species tested. The protocol has been validated for thousands of DNA samples by generating high data quality in dense SNP arrays. DNA extracted from *Eucalyptus* spp. leaf and cambium as well as mycelium from *Trichoderma* spp. was readily digested with restriction enzymes and performed consistently in AFLP assays. Scaled-up DNA extractions were also suitable for long read sequencing. Successful RNA quality control and good RNA-Seq data for *Eucalyptus* and cashew confirms the effectiveness of the sorbitol buffer pre-wash for high quality RNA extraction.

## Introduction

Plant DNA extraction protocols are often reported for only one or a few species, or are suitable only for specific applications [1–4]. In our experience, commercial kits, expensive for large-scale projects, are frequently inefficient for DNA extraction from challenging plant tissues rich in polyphenols or polysaccharides. Protocols requiring special items such as magnetic beads or binding columns [5,6] may not be easily accessible or are prohibitively expensive in many countries. In our laboratory, the classic CTAB procedure [7] has been used successfully for a wide variety of plants, although for some species, the DNA obtained has not provided consistent results. This has become a significant problem for newer applications, such as high-throughput SNP genotyping, short-read Illumina sequencing and single molecule sequencing assays that require unfailing input DNA. The most common interfering metabolites from plant tissues in molecular assays are DNA-bound oxidized phenolics and co-precipitated polysaccharides [8–13]. A high salt CTAB extraction buffer, frequently used to mitigate polysaccharide contamination [14], proved ineffective for many species in our hands. SDS-based protocols are frequently cited as an alternative to CTAB, but these may be suitable for a narrower range of species or tissue types [15].

In the search for an economical protocol for plant DNA extraction that we could apply universally, we adopted a modification of a relatively simple high salt CTAB protocol that, by using a pre-wash with a sorbitol-containing buffer, aims to remove interfering contaminants from tissue macerates prior to cell lysis. A sorbitol pre-wash step combined with a regular CTAB extraction protocol was previously described to extract DNA from leaf tissue of several different plant species [16–20]. Nevertheless, the suitability of the technique for high-throughput sample processing and its applicability to different plant tissues, such as cambium has not been tested, nor its utility for DNA extraction from fungal mycelium. Herein we report a versatile and optimized DNA extraction protocol using a sorbitol pre-wash, combined with high salt CTAB and its application in ten plant genera, representing nine families. We also report on the successful application of our protocol for extraction of DNA from a collection of isolates of filamentous fungi in the genus *Trichoderma*, mostly belonging to the *Trichoderma harzianum* species complex (Hypocreaceae). All species tested were subjects of ongoing studies conducted by our group, where we have previously experienced difficulties in obtaining high quality DNA due to high fiber, polysaccharide or polyphenol content. This relatively simple, rapid and economical protocol, applicable to a variety of demanding high-throughput applications, provides significantly improved DNA quality, purity and consistency across phylogenetically diverse species and in sample types that include leaves, cambium and fungal mycelium. In addition, we report for the first time on the utility of a sorbitol pre-wash for plant RNA extraction in various tissues of *Eucalyptus grandis* (leaves and developing cambium) and cashew (*Anarcardium occidentale*) (leaf, flower, stem, fruit, pseudofruit and embryo).

## Materials and methods

### Standard DNA extraction protocol

#### Sample grinding

Effective tissue maceration is critical for good DNA yield. While mortar and pestle grinding with liquid nitrogen is optimal, the ability to process many samples quickly and simultaneously, is generally a prerequisite. The quantity of tissue used for a single tube mini-prep should be between 100–150 mg of fresh tissue or approximately 20–30 mg or 2 cm^2^ in the case of silica gel or air-dried leaf material. Among the methods we tested, the most efficient one used prior lyophilization of fresh material, including fungal mycelium, in 2.0 ml microtubes. Two to three 20-second cycles of maceration in a bead mill (beadbeater, Model 1001, Biospec Products, Bartlesville, OK, USA) with approximately seven to ten 2.45 mm AISI 316 stainless steel ball bearings (measured using an improvised scoop), were usually sufficient to reduce dry samples to a fine powder. An alternative to lyophilization is freezing microtubes containing fresh tissue and ball bearings, along with the bead mill block, at -80°C, prior to maceration. Hard materials such as wood fragments still require freezing in liquid nitrogen. All of these methods could be scaled to a 96 x 1.2 ml polypropylene cluster tube format, if appropriate centrifuge and rotor are available.

#### Sorbitol pre-wash

The sorbitol wash buffer (100 mM Tris-HCl pH 8.0, 0.35 M Sorbitol, 5 mM EDTA pH 8.0, 1% (w/v) Polyvinylpyrrolidone (average molecular weight 40,000; PVP-40)) was made ready for use by the addition of 2-mercaptoethanol (1% v/v) just before extraction. The buffer base may be stored at 4°C, for up to six months. An excess of sorbitol wash buffer, sufficient to fill sample tubes containing macerated plant material to approximately ¾ capacity (0.9–1.5 ml, depending on tubes used), was added. Tubes were capped and shaken for five seconds in the bead mill, shaken manually or mixed using a vortex. Tubes were inspected to confirm suspension of the powdered material and mixed again, if necessary. Samples were then centrifuged at 2,500–5,000 x g for five minutes at room temperature, where we found that 5,000 x g was the upper limit to avoid deformation or rupture of polypropylene tubes containing ball bearings. Different brands of tube should be tested empirically for resistance in pilot extractions. Following centrifugation, supernatants containing polysaccharides and polyphenols were carefully decanted or aspirated and discarded. For most species we have tested, we usually find the first supernatant to be lightly cloudy or clear and tan to light brown in color. In these samples, a single sorbitol pre-wash is sufficient. The sorbitol wash may be repeated, however, for especially challenging samples where the supernatant from the first wash is found to be viscous, densely turbid or dark brown in color. The pre-wash step(s) only adds 10–20 minutes to the standard CTAB protocol for a batch of 32 samples in 1.5 or 2.0 ml microtubes or a 96-well cluster tube array.

#### Sample lysis and extraction

High salt CTAB lysis buffer, containing 100 mM Tris-HCl pH 8.0, 3 M NaCl, 3% CTAB (cetyl trimethylammonium bromide), 20 mM EDTA and 1% (w/v) polyvinylpyrrolidone (PVP-40; average molecular weight 40,000), may be stored at room temperature, for up to six months. This lysis buffer is made ready for use by the addition of 2-mercaptoethanol (1% v/v) before extraction and pre-warmed to 65°C to aid pipetting. The pre-warmed lysis buffer was added to the sample tubes (500 to 700 μl or to approximately ½ the sample tube capacity) and the samples resuspended by shaking for five seconds in the bead mill or by vortexing. The ball bearings remaining in the tubes greatly assist the macerate mixing process. Tubes were then incubated in a water bath, oven or hot block at 65°C for a minimum of 30 minutes, up to 60 minutes, with mixing by inversion every ten minutes. Samples were then cooled at room temperature for five minutes. A volume of chloroform:isoamyl alcohol (24:1 v/v; CIA), approximately equal to the lysis buffer, was added to the sample tubes, which were then shaken vigorously or vortexed for 10 seconds. This can be efficiently accomplished using the bead mill if desired. Samples were centrifuged at 2,500–5,000 x g for ten minutes at room temperature. The upper aqueous phase was carefully transferred to a new tube by pipetting, carefully avoiding disturbance of the debris between phases. Following extraction and centrifugation of the lysates, the aqueous phase was usually colorless or light tan. Although usually unnecessary, the CIA extraction can optionally be repeated with centrifugation at up to 13,000 x g (without ball-bearings) for 10 minutes and recovery of the upper phase to a fresh tube. Nucleic acids were precipitated from the recovered upper phase by the addition of 0.1 times its volume of 3 M sodium acetate pH 5.2 and 0.66 times its volume of cold isopropanol (stored at -20°C). Tubes were mixed by inversion and kept at -20°C for one hour. DNA was pelleted by centrifugation at 13,000 x g for 10 minutes at room temperature or, in case of 96 well cluster tube arrays, at the maximum allowable speed for the plate rotor. The supernatants were carefully decanted off and tubes drained by resting inverted on paper towels. Pellets were washed by the addition of 0.7–1 ml of 70% ethanol and tubes centrifuged for 10 minutes, as before. Supernatants were carefully removed by aspiration to avoid loss of the nucleic acid pellet and tubes left to dry open at room temperature for approximately one hour or vacuum dried at room temperature for 10 minutes. DNA pellets following isopropanol precipitation were usually small, compact, translucent and only rarely brownish, indicating that the pre-wash effectively removes polysaccharides and polyphenols. Pellets were then suspended in 100 μl TE containing 0.1 mg ml^-1^ DNase-free RNase A and incubated at 37°C for 30 minutes. The extracted DNA was stored at -20°C until required.

#### DNA quality evaluation

DNA purity was estimated using a spectrophotometer, (Nanodrop 2000; Thermo Fisher Scientific). DNA yield was estimated, using a fluorimeter and fluorescent DNA-binding dye (Qubit^TM^ dsDNA BR Assay Kit; Thermo Fisher Scientific), according to the manufacturer´s instructions. DNA integrity was checked by agarose gel electrophoresis.

To test the effectiveness of the reported DNA extraction protocol, we performed a series of small-scale parallel DNA extractions, with and without the sorbitol pre-wash step. Leaves of several plant genera regarded as “demanding” in our laboratory were tested, including *A*. *occidentale* (Anacardiaceae) (Cashew), *E*. *grandis* (Myrtaceae), *Pereskia aculeata* (Cactaceae), several different species of the genera *Diplusodon* and *Lafoensia* (both Lythraceae). DNA yields and purity were estimated spectrophotometrically. DNA quality was also inferred by a simple PCR amplification assay using the nuclear ribosomal ITS marker. Each reaction contained 1 X PCR buffer with 2.0 mM MgSO_4_, 0.2 mM dNTP’s, 0.2 M Trehalose, 0.3 μM each of the universal primers An5 and An4 [21], 1 U Taq DNA polymerase and 1 μl undiluted DNA. PCR cycling consisted of two minutes initial denaturation at 95°C then 35 cycles of 20 seconds at 95°C, 40 seconds at 55°C and 80 seconds at 72°C, followed by 7 minutes at 72°C. PCR products were analyzed using an ethidium bromide stained 1.5% w/v agarose gel, where the expected band size of the ITS fragment was approximately 650 bp.

### Method variations

For applications requiring high yields and especially high molecular weight genomic DNA, such as long read single molecule PacBio sequencing (Pacific Biosciences, CA, USA), several grams of fresh tissue may be macerated with a mortar and pestle in the presence of liquid nitrogen. The extraction protocol is scaled up to use large 15-ml or 50-ml tubes or several microtubes in parallel and the DNA consolidated at the end. Gentle handling must be adopted throughout the entire procedure, using only slow vortexing and careful pipetting, preferably using wide-bore tips, including the resuspension of the tissue macerate following the sorbitol pre-washes. If the final DNA precipitate forms a visible clump, it should be recovered using a hook fabricated from a glass Pasteur pipette and transferred to a fresh tube to be washed with 70% ethanol. Otherwise DNA may be recovered by centrifugation for five minutes at 5,000 x g and washed with 70% ethanol. Following air drying for one hour at room temperature in an open tube, the DNA may be gently resuspended in an appropriate volume of water or buffer compatible with the intended downstream analysis. DNA integrity can be estimated using pulsed-field gel electrophoresis and the adequate fraction selected for downstream sequencing using the Blue pippin DNA size selection system (BluePippin; Sage Science, Beverly, MA, USA).

### RNA extraction protocol

For RNA extraction, the biological material was kept frozen at all times until the addition of sorbitol buffer. It is possible to freeze tubes containing samples and ball bearings at -80°C along with the bead mill sample block, before using the bead mill, but samples are frequently found to have thawed by the end of maceration. We also tried freezing tubes in liquid nitrogen before using the bead mill, but frequently lost samples because of broken tubes. If high throughput is not an issue, we prefer to macerate tissues using a mortar and pestle in liquid nitrogen for RNA extraction. Making sure to keep the tissue frozen with either liquid N_2_ or dry ice, macerated samples were transferred into 2.0 ml tubes. Immediately, an excess of sorbitol wash buffer with 1% 2-mercaptoethanol (v/v) was added to fill sample tubes to approximately ¾ capacity (0.9–1.5 ml, depending on tubes used). Tubes were capped and shaken in the bead mill, mixed using a vortex or manually. Tubes were inspected to confirm suspension of the powdered material and shaken again, if necessary. Tubes were then centrifuged at 2,500 x g for five minutes at room temperature, supernatants aspirated from samples and discarded. We routinely used two rounds of the sorbitol solution wash for RNA extraction.

RNA extraction is then continued, essentially as previously described [22]. High salt CTAB extraction buffer was made ready for use by the addition of 2% (v/v) 2-mercaptoethanol just before extraction and pre-warmed to 65°C. For minipreps in 2 ml microtubes, 1.0 ml of pre-warmed CTAB extraction buffer was quickly added. Tubes were then shaken vigorously and placed in a 65°C water bath for at least 20 minutes. The amount of tissue used for a single miniprep is dependent on type, we routinely use approximately 100 mg for fresh leaves and 80 mg for cambium.

About 750 μl of chloroform was added, tubes shaken vigorously and centrifuged for 10 minutes at 12,000 x g. The upper aqueous phase was carefully aspirated, transferred to a fresh tube and the chloroform extraction repeated. The upper aqueous phase was again carefully transferred to a fresh tube, placed on ice and an equal volume of a 7.5 M LiCl, 50 mM EDTA solution added. Following mixing by inversion, RNA was precipitated for two to four hours at -20°C and pelleted by centrifugation at 12,000 x g for 20 minutes at 4°C. Alternatively, precipitation can take place overnight at 4°C, but in our experience, there is usually no significant gain in yield. The supernatant was carefully removed and discarded, taking care not to lose the pellet, which was then dissolved in 500 μl SSTE. Samples were then extracted once with 500 μl chloroform, shaking or vortexing before centrifuging at full speed for ten minutes. The aqueous phase was transferred to a new microtube, two times its volume of 95% ethanol added and, following mixing, the RNA allowed to precipitate for a minimum of 15 minutes at -80°C. RNA was then pelleted by centrifugation at full speed for 20 minutes at 4°C. The supernatant was discarded, being careful not to lose the pellet, which was then washed with 200 μl cold 70% ethanol and centrifuged at full speed for five minutes. The supernatant was carefully aspirated. Pellets were then allowed to air dry, for 5 to 10 minutes and dissolved in RNase free water.

The protocols contained in this paper have been deposited, in detailed form, at protocols.io: http://dx.doi.org/10.17504/protocols.io.tzfep3n.

## Results and discussion

**Fig 1:**
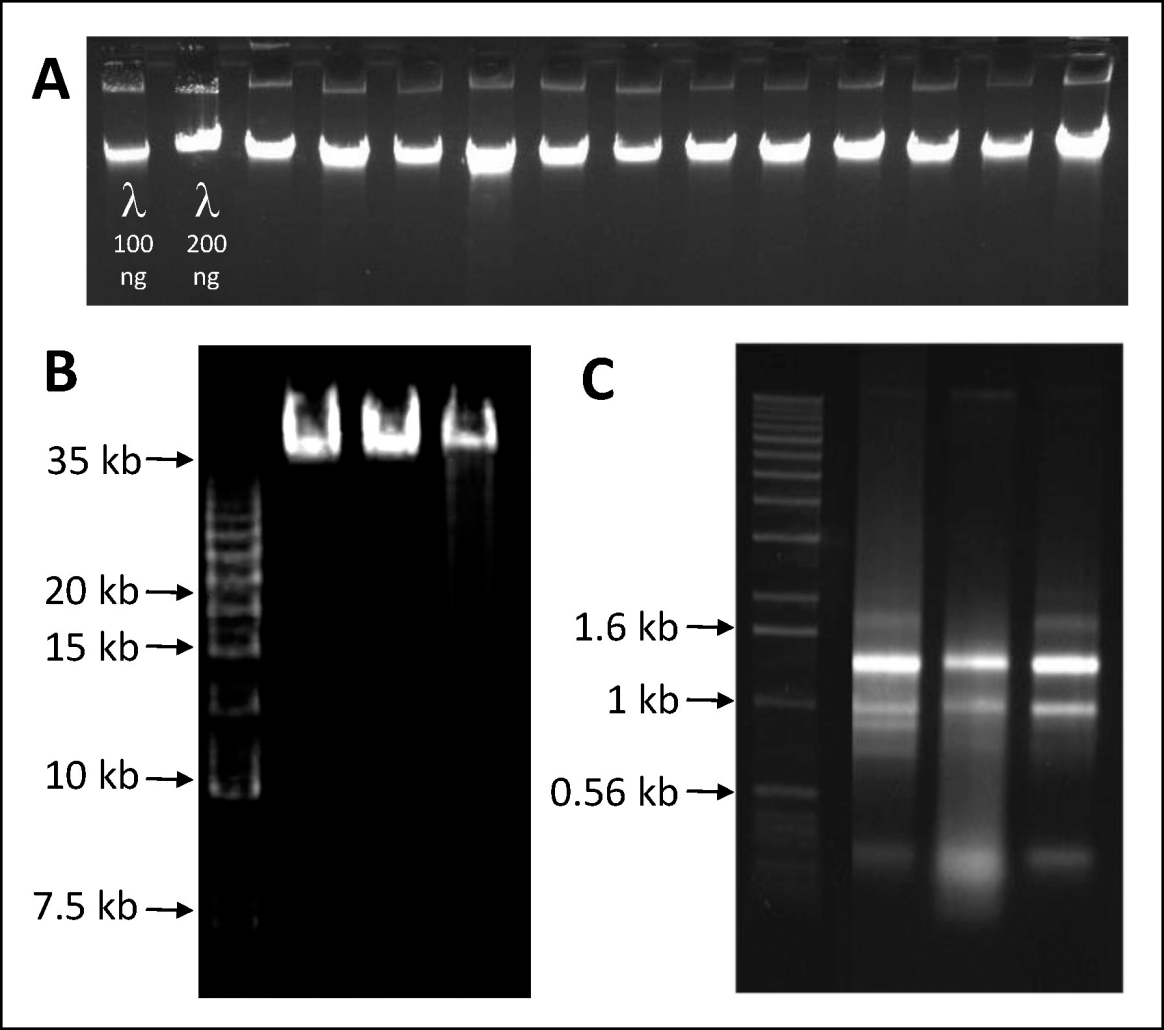
Gel images of nucleic acids extracted using the described protocol. (A) Electrophoresis (1% agarose-TBE) of genomic DNA extracted from *Eucalyptus grandis* (lanes 3–8) and cashew (*Anacardium occidentale*) (lanes 9–14) leaves; lanes 1 and 2 contain 100 and 200 ng of uncut Lambda DNA (Thermo Scientific), respectively; (B) Pippin pulse (Sage Science, Beverly, MA, USA) gel electrophoresis (0.75% agarose-KBB buffer) image provided by McGill/Genome Quebec Innovation Centre with cashew high molecular weight genomic DNA samples; lane 1 is the 2.5 kb molecular ruler (BioRad Laboratories); (C) Electrophoresis (1% agarose-TAE) of total RNA from *E*. *grandis* leaf, fruit bud and cambium (lanes 2–4); lane 1 is kb Plus DNA ladder (Thermo Scientific).

DNA extracted from leaves or cambium of eight genera of plants and mycelium of one fungal genus consistently produced 260/280 absorbance ratios of 1.8 or higher (Table 1), which indicates high purity regarding protein contamination [23] and was undegraded, as estimated by agarose gel electrophoresis (Fig 1A). Similarly, 260/230 absorbance ratios were usually between 2.05 and 2.40, indicating very low levels of polyssacharide contamination [23]. Double-stranded DNA yields from single microtube minipreps, as estimated by Qubit^TM^ (ThermoFisher Scientific) fluorimeter, were in the range of 1.0 to several micrograms, fully adequate for the majority of downstream molecular applications (Table 1). The average 260/230 ratio for *Eucalyptus* spp. cambium DNA samples was lower than ideal at 1.52–1.74 suggesting some residual impurity. However, the DNA was colorless and performed well in downstream analyses such as genotyping on an Infinium 60K SNP chip (Illumina) [24]. High quality, mucilage-free DNA was also recovered from fleshy leaves of *Pereskia aculeata*, a member of the Cactaceae, notorious for their high mucilage content [18]. Several hundred samples of DNA from fresh or dried leaf samples of coffee (*Coffea canephora*), cassava (*Manihot esculenta*), cashew (*Anacardium occidentale*) and cambium of the Brazilian pine (*Araucaria angustifolia*) were successfully genotyped with an Axiom (Affymetrix) SNP array with sample call rates above 90% [25,26]. High quality data were also obtained for *Eucalyptus* leaf samples genotyped by sequence-based genotyping methods such as RAD-sequencing [27] and DArT-sequencing [28]. *Eucalyptus* leaf and cambium DNA was readily digested by restriction enzymes, as indicated by consistent performance in methylation sensitive amplification polymorphism analysis (MSAP) [29] (S1 Fig). *Trichoderma* DNA also performed reliably in AFLP assays and in PCR amplification of several different loci used for sequence-based identification, including nuclear ribosomal ITS, RNA-polymerase II gene (*RPB2*), calmodulin and translation elongation factor 1-α (*TEF1α*) [30]. Using these data, we were able to identify the *Trichoderma* isolates as several different members of the *Trichoderma harzianum* species complex, *Trichoderma afroharzianum*, *Trichoderma spirale*, *Trichoderma koningiopsis*, *Trichoderma hamatum*, *Trichoderma longibrachiatum* and *Trichoderma asperelloides* (P. Inglis, unpublished). It is likely that our sorbitol pre-wash + CTAB DNA extraction protocol is also applicable to many other fungi, at least within the Ascomycota.

**Table 1:** Purity and yield of DNA extracted with the described protocol from various plant species and tissue types.

Species (Family)	Tissue	n	Mean yield (μg ± SD)^a^	Mean A_260_/A_280_^b^	Mean A_260_/A_280_^b^
*Eucalyptus* spp. (Myrtaceae)†	Leaf	185	7.4 ± 5.6	2.02	2.40
*Eucalyptus* spp. (Myrtaceae) †	Cambium	36	1.6 ± 0.7	2.06	1.52
*Anacardium occidentale* (Anacardiaceae)*	Leaf	1152	4.52 ± 1.8	1.97	2.15
*Pereskia aculeata* (Cactaceae)	Leaf	92	5.4 ± 2.2	2.11	2.31
*Manihot esculenta* (Euphorbiaceae)*	Leaf	576	47.0 ± 20.0	1.99	2.00
*Arachis* spp. (Fabaceae)†	Leaf	92	3.4 ± 3.5	2.06	2.24
*Coffea canephora (*Rubiaceae)*	Leaf	1536	12.24 ± 9.8	1.96	2.12
*Araucaria angustifolia* (*Araucariaceae*)*	Cambium	545	4.27 ± 1.8	1.85	1.74
*Zea mays* (Poaceae)	Leaf	96	11.5 ± 4.9	1.90	2.02
*Trichoderma* spp. (Hypocreaceae–Fungi) †	Mycelium	250	6.3 ± 4.8	2.05	2.17

^a^Mean yield was estimated from Qubit dsDNA BR Assay Kit (Thermo Scientific) readings considering the DNA resuspension volume for each single preparation starting from approximately 100–200 mg of fresh or 20–40 mg of lyophilized or air-dried tissue.

^b^Mean UV absorbance ratios were estimated from Nanodrop 2000 readings.

* DNA extracted using a 96 x 1.2 ml cluster tube format.

†Mixed collection of species or hybrids from phylogenetic or comparative studies.

High molecular weight genomic DNA was extracted from cashew leaf samples treated with the protocol variation using mortar and pestle maceration, gentle handling and DNA recovery with a glass hook. This DNA was evaluated by PFGE (Pulsed Field Gel Electrophoresis) at the MacGill/Genome Quebec facility as being >35kb in size (Fig 1B) and was used for PacBio sequencing in 42 SMRT (Single Molecule Real Time) cells, providing 27 Gb of filtered data in 3.08 M reads with an N50 of 12.2 kb [31].

In our small scale parallel extraction experiments, we obtained generally higher yields of purer DNA using the sorbitol pre-wash (Table 2), with the exception of *Diplusodon* spp., where a slightly lower yield was obtained. The most extreme benefit of the pre-wash was obtained in cashew, where we obtained an almost three-fold increase in DNA yield.

**Table 2:** Purity and yield of DNA extracted from leaves of five different plant genera using the reported CTAB-based protocol with and without two sorbitol pre-washes.

			Standard CTAB miniprep			CTAB + Sorbitol Pre-wash		
Species (Family)	Storage	n (pairs)	Yield (ug)^a^	Mean A_260_/A_280_^b^	Mean A_260_/A_280_ ^b^	Yield (ug)^a^	Mean A_260_/A_280_ ^b^	Mean A_260_/A_280_ ^b^
*Anacardium occidentale* (Anacardiaceae)	Fresh	5	10.54	1.898	1.186	32.84	1.96	1.758
*Diplusodon* spp. (Lythraceae)	Silica gel	5	9.822	1.67	1.562	7.573	2.06	1.518
*Eucalyptus grandis* (Myrtaceae)	Dried 4°C	10	16.706	2.095	2.395	25.868	2.173	2.312
*Lafoensia* spp. (Lythraceae)	Herbarium	8	8.317	1.296	1.645	13.194	2.035	2.049
*Pereskia aculeata* (Cactaceae)	-80°C	12	10.747	1.803	1.418	18.375	2.056	2.012
Mean			11.226	1.752	1.641	19.57	2.057	1.93

^a^Mean yield was estimated from Qubit dsDNA BR Assay Kit (Thermo Scientific) readings considering the DNA resuspension volume for each single preparation starting from approximately 100–200 mg of fresh or frozen tissue or 20–40 mg of dry tissue.

^b^ Mean UV absorbance ratios were estimated from Nanodrop 2000 readings.

**Fig 2:**
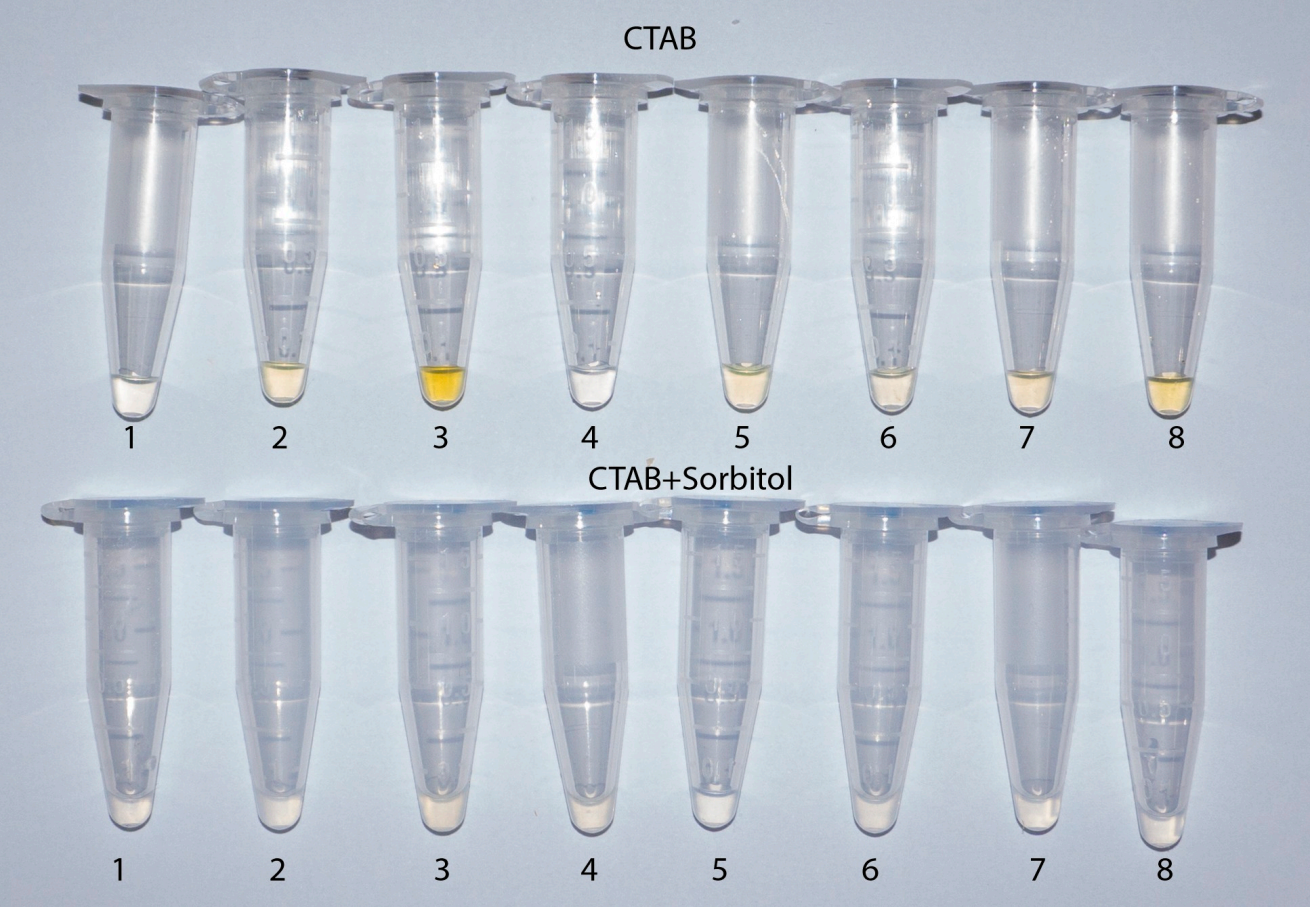
Resuspended DNA samples extracted from *Lafoensia* spp. The top row of tubes contains samples extracted using our standard CTAB protocol and the bottom row contains paired samples extracted using the same protocol with the addition of two sorbitol solution pre-washes.

**Fig 3:**
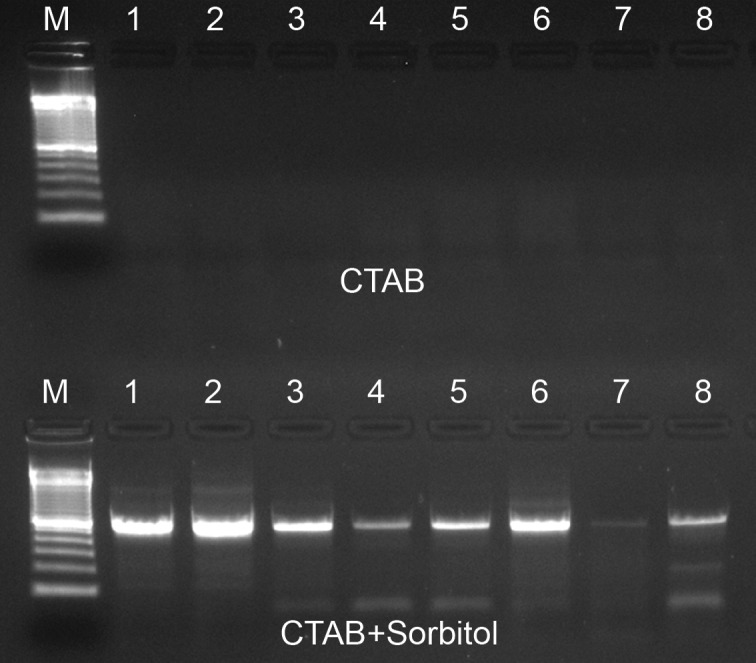
ITS PCR of *Lafoensia* spp. 1.5% agarose gel containing PCR products from reactions performed using either standard CTAB extracted DNA (top row of wells) and paired samples extracted using the same protocol with the addition of two sorbitol solution pre-washes (bottom row). The molecular marker is the 100 bp ladder (Invitrogen).

**Fig 4:**
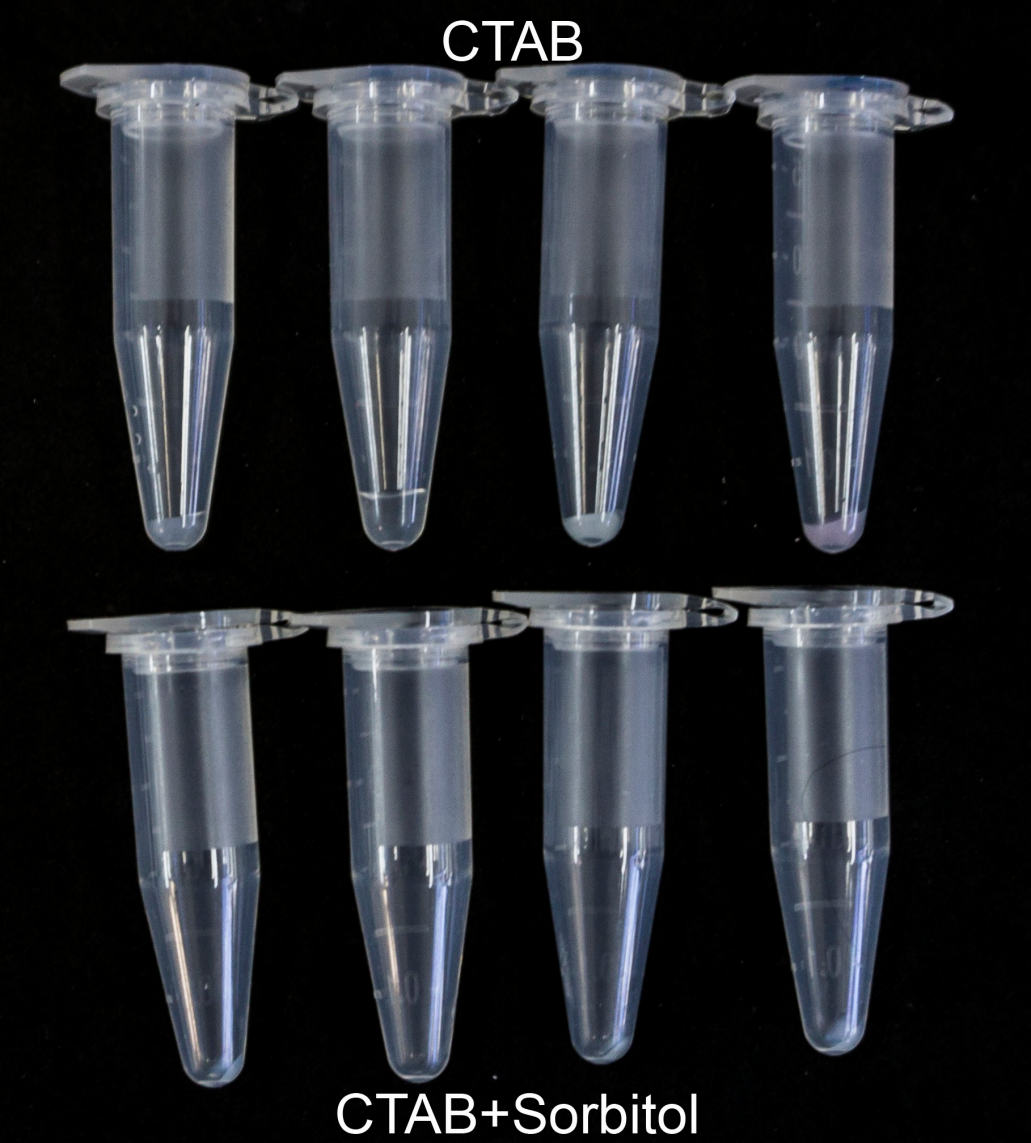
Pellets formed following centrifugation of isopropanol-precipitated DNA extracts from *Pereskia aculeata*. The top row of tubes contains samples extracted using our standard CTAB protocol and the bottom row contains paired samples extracted using the same protocol with the addition of two sorbitol solution pre-washes.

An especially large improvement in DNA purity was notable from herbarium leaf specimens of *Lafoensia* spp. where, without sorbitol pre-washing, several extracted DNA samples were yellow or brown colored, probably indicating contamination with oxidized polyphenols (Fig 2). Even more dramatically, ITS PCR failed in all of the *Lafoensia* spp. samples extracted with standard CTAB protocol, which were effectively rescued by the sorbitol pre-wash (Fig 3), but not by ten-fold DNA dilution in water prior to PCR (not shown). In other parallel test samples, however, the ITS marker amplified easily both with and without the pre-wash step (not shown). This was particularly surprising in the case of *Pereskia aculeata*, where polysaccharide contamination in samples without pre-wash manifested as extremely large gelatinous pellets following isopropanol precipitation and centrifugation (Fig 4), making resuspension and pipetting difficult. This amplification success can probably be explained by the fact that polysaccharides are diverse in their capacity to inhibit PCR [13]. Several neutral polysaccharides, including arabinogalactan, dextran, gum guar, gum locust bean, inulin, mannan and starch were shown not to be PCR inhibitory, whereas many acidic polysaccharides, such as carrageenan, dextran sulfate, gum ghatti, gum karaya, pectin, and xylan were strongly inhibitory [32].

A far more intractable problem for downstream molecular manipulations is often encountered in samples rich in oxidized polyphenols, which are thought to become irreversibly bound to DNA [33,34]. Our CTAB lysis buffer contains a high salt content, which has been recommended and is widely used for plants rich in polysaccharides, 2-mercaptoethanol to inhibit polyphenol oxidation and polyvinylpyrrolidone (PVP-40) to bind and remove polyphenols [35]. However, we have not found these modifications to be effective in many of the plant species or sample types we have handled, in contrast to the wide applicability of sample pre-washing methodology. This is probably related to its supposed mechanism of action, where nucleic acids are kept out of solution in the presence of 0.35 M sorbitol [14], while the pre-washes act to reduce or remove polyphenols before they can covalently bind to DNA and reduce polysaccharides before they can be co-extracted and later co-precipitated by alcohol, along with the nucleic acids.

The sorbitol prewash extraction protocol produces high DNA yields and purity estimates regardless of tissue storage method (Tables 1 and 2), which appear to be more closely dependent on sample taxonomy, developmental age or tissue type, regardless of tissue storage method (Tables 1 and 2). Tissue storage though is likely to have a strong influence on DNA integrity, which we only estimated in some -80°C frozen *E*. *grandis* and *A*. *occidentale* samples (Fig 1A) and in three *A*. *occidentale* samples subjected to special handling required for long-read sequencing (Fig 1B). The most fragmented and compromised DNA is expected from herbarium material, where sample drying is commonly accomplished at high temperatures (60–70°C), resulting not only in double stranded breaks, but also in inter-strand crosslinks, abasic sites or other structural modifications which further accumulate over time [36]. Heterogeneously damaged DNA from herbarium material among our test samples is the likely explanation for the strongly variable band intensity observed in the *Lafoensia* spp. ITS PCR results (Fig 3). Although tested in only one genus, the sorbitol pre-wash technique appears to be highly suitable for DNA extraction from herbarium specimens.

We successfully integrated the sorbitol pre-wash into our RNA extraction protocol. High yields of high quality RNA were obtained from different tissues of *Eucalyptus* and cashew (Fig 1C), species particularly rich in polyphenols and polysaccharides (Table 3).Samples were successfully used for high throughput sequencing using Illumina HiSeq, generating supporting data for genome annotation of both species [31,37]. RNA sequencing data for *Eucalyptus* are available at Phytozome (https://phytozome.jgi.doe.gov/pz/portal.html-!info?alias=Org_Egrandis).

**Table 3:** Purity and yield of extracted RNA with the described protocol from two plant species and different tissue types rich in secondary metabolites and polysaccharides.

Species (Family)	Tissue type	n	Mean RNA yield (μg ± SD)^a^	Mean A_260_/A_280_^b^	Mean A_260_/A_280_^b^
*Eucalyptus grandis* (Myrtaceae)	Juvenile leaf	3	1.43 ± 0.35	2.14	2.36
	Developed leaf	3	1.30 ± 0.10	2.17	2.31
	Cambium	3	1.30 ± 0.44	2.00	2.43
*Anacardium occidentale* (Anacardiaceae)	Leaf	2	2.40 ± 0.00	2.17	2.29
	Flower	2	2.42 ± 0.59	2.14	2.27
	Stem	2	1.06 ± 0.30	2.10	2.18
	Fruit	1	0.83	2.03	2.27
	Pseudofruit	2	1.03 ± 0.15	2.07	2.20
	Embryo	1	1.05	2.01	2.36

^a^ Mean yield was estimated from Qubit RNA BR Assay Kit (Thermo Scientific) readings considering the RNA resuspension volume for each single preparation starting from approximately 100–200 mg of fresh tissue.

^b^ Mean UV absorbance ratios were estimated from Nanodrop 2000 readings.

Although addition of the sorbitol pre-wash step extends the time required for a standard DNA or RNA extraction protocol by up to half an hour, we found that the downstream benefits of high nucleic acid purity, quality and yield leads to improved reliability and consistency of assays and avoidance of failed reactions for a variety of species, which more than compensates for this extra time. Additional reagent and plasticware costs are low.

## Conclusions

In conclusion, we show that pre-washing of sample macerates with buffered sorbitol solution prior to DNA extraction with a CTAB-based protocol enables good yields and consistent quality of high molecular weight DNA from ten plant genera and mycelium from several species in the ascomycete fungal genus *Trichoderma*. The extracted DNA is suitable for a range of high-throughput molecular assays such as SNP genotyping and high-throughput short-read Illumina sequencing as well as long read PacBio sequencing. Optimal results and ease of handling in plants are obtained for either fresh or dried leaf tissue, but the protocol is also efficient for cambium and fruit tissue and is likely to be applicable generally. We also show, for the first time, that a sorbitol pre-wash may be added to a protocol for the extraction of high quality RNA from plants. Detailed protocols are provided (S1 File).

## Supporting information

S1 Fig Example MSAP profiles. Pseudogel image generated by Genographer (v. 2.0, available at https://sourceforge.net/projects/genographer/) of fluorescent methylation-sensitive AFLP profiles using *Eucalyptus grandis* DNA. Isogenic samples were derived from mature leaf, juvenile leaf and cambium and were digested with either EcoRI + MspI (sample suffix M) or EcoRI + HpaII (sample suffix H) prior to adapter ligation and primary and secondary AFLP PCRs.
(TIF)

S1 File Detailed protocols for DNA and RNA extractions. (DOCX)
