# Supplementary figures and images for: Fast and inexpensive protocols for consistent extraction of high quality DNA and RNA from challenging plant and fungal samples for high-throughput SNP genotyping and sequencing applications
Source: PLoS One. 2018 Oct 18;13(10):e0206085. doi: 10.1371/journal.pone.0206085 (PMC6193717; doi:10.1371/journal.pone.0206085)

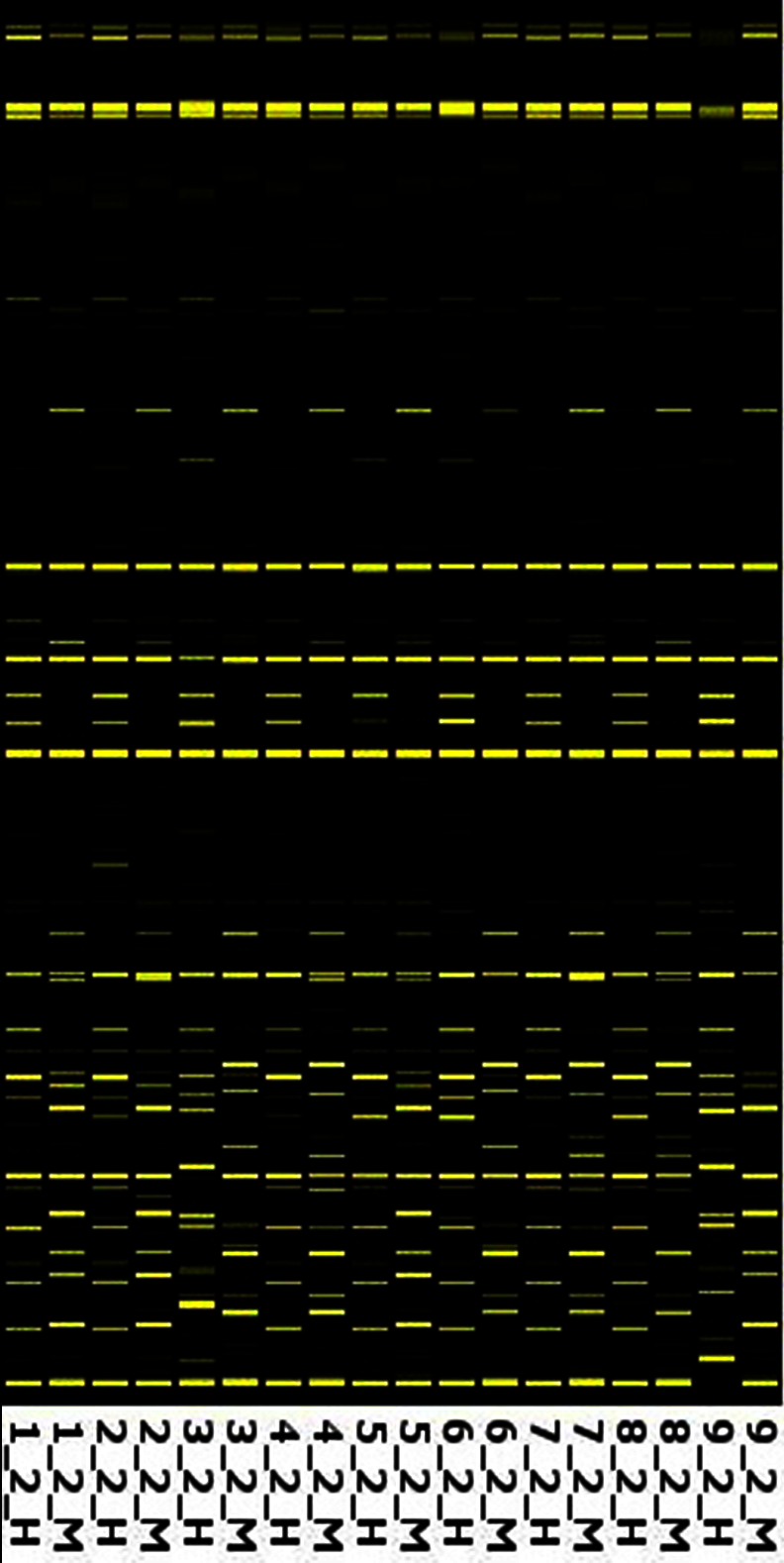

Supplement: S1 Fig — Pseudogel image generated by Genographer (v. 2.0, available at https://sourceforge.net/projects/genographer/) of fluorescent methylation-sensitive AFLP profiles using Eucalyptus grandis DNA. Isogenic samples were derived from mature leaf, juvenile leaf and cambium and were digested with either EcoRI + MspI (sample suffix M) or EcoRI + HpaII (sample suffix H) prior to adapter ligation and primary and secondary AFLP PCRs. (TIF) [file pone.0206085.s001.tif]
