# Supplementary material for: Fast and inexpensive protocols for consistent extraction of high quality DNA and RNA from challenging plant and fungal samples for high-throughput SNP genotyping and sequencing applications
Source: PLoS One. 2018 Oct 18;13(10):e0206085. doi: 10.1371/journal.pone.0206085 (PMC6193717; doi:10.1371/journal.pone.0206085)
Supplement: S1 File — (DOCX) [file pone.0206085.s002.docx]

# **Fast and inexpensive protocols for consistent extraction of high quality DNA and RNA from challenging plant and fungal samples for high-throughput SNP genotyping and sequencing applications: Detailed Protocols**

Peter W. Inglis^1^, Marilia de Castro R. Pappas^1^, Lucileide V. Resende^1^, Dario Grattapaglia^1,2^

^1^Plant Genetics Laboratory, Embrapa Genetic Resources and Biotechnology, Brasília, Brazil;

^2^Genomic Sciences and Biotechnology Program, Universidade Católica de Brasília, Brasília, Brazil

## Reagents:

## Solutions for DNA extraction

**Sorbitol wash buffer**: 100 mM Tris-HCl pH 8.0, 0.35 M Sorbitol, 5 mM EDTA pH 8.0, 1 % (w/v) Polyvinylpyrrolidone Molecular wt. 40,000 (PVP-40). This buffer base may be stored at 4°C, for up to six months. The wash buffer is made ready for use by the addition of 2-mercaptoethanol (1 % v/v) before the extraction.

**Extraction buffer**: 100 mM Tris-HCl pH 8.0, 3 M NaCl, 3% CTAB (cetyl trimethylammonium bromide), 20 mM EDTA, 1% (w/v) Polyvinylpyrrolidone. This buffer is stored at room temperature, for up to six months. The lysis buffer is made ready for use by the addition of 2-mercaptoethanol (1 % v/v) before the extraction.

**CIA**: 24:1 (v/v) mixture of chloroform:isoamyl alcohol.

## Solutions for RNA extraction

**CTAB extraction buffer**: 2 % CTAB, 2 % polyvinylpyrrolidone (PVP; 40,000), 100 mM Tris-HCl pH 8.0, 25 mM EDTA, 2 M NaCl, 0.5 g/L spermidine. Autoclave the solution and add 2-mercaptoethanol (2 % v/v) immediately before use.

**SSTE**: 1.0 M NaCl**,** 0.5 % SDS**,** 10 mM Tris-HCl pH 8.0**,** 1 mM EDTA pH 8.0**.**

**LiCl_2_: EDTA**: 7.5 M LiCl_2_, 50 mM EDTA. This solution needs to be filter sterilized to remove particulates**.**

## Procedure

**Biological material**: Optimal and most predictable DNA extraction results are obtained using leaf tissue, but the protocols may also be applied successfully to other tissues such as cambium, fruit tissue and fungal mycelium (conveniently scraped from the surface of solid growth medium). The quantity of tissue used for a single tube mini-prep should be between 100-150 mg of fresh tissue or approximately 20-30 mg or 2 cm^2^ in the case of herbarium-stored or silica gel or air-dried leaf material.

We observe that the initial amount of biological material is more flexible than that for conventional extraction protocols and can be doubled or tripled, often greatly increasing yield whilst not affecting purity. The sorbitol pre-wash appears to improve the effectiveness of the extraction buffer, preventing it from becoming saturated.

**Sample maceration**: Efficient maceration of biological material is critical for good DNA yield. Mortar and pestle grinding in the presence of liquid nitrogen is effective but is impractical when the aim is to process many samples simultaneously. Various maceration methods were investigated, but the most consistent and effective one was prior lyophilization of fresh material for at least two hours. For tender leaf samples, this could be substituted by either dehydration for seven days in a standard refrigerator, sample storage with silica gel packing or simply be bypassed in the case of herbarium material. For small scale extractions, material may be dried in open 2.0 ml microtubes (brands previously tested for resistance to the action of the bead mill). For large-scale extractions, standard 96 well format tube racks fitted with strips of eight or twelve 1.1 ml or 1.2 ml polypropylene tubes are used. Approximately seven to ten 2.45 mm AISI 316 stainless steel ball bearings are added to each tube. To facilitate this, we improvised a scoop from a 200 µl PCR microtube pierced by a metal rod (Figure 1). Two to three 20-second cycles of maceration in a bead mill (beadbeater, Model 1001, Biospec Products, Bartlesville, OK, USA) were usually sufficient to reduce samples to a fine powder. Similar tissue homogenizers such as Fastprep (MP Biomedicals), Genogrinder, Mini-G (SPEX SamplePrep) or TissueLyser (Qiagen) work equally well. Fresh samples can be processed in a similar manner if previously frozen at -80 °C, together with the sample block of the bead mill. Occasionally, particularly resistant materials may require freezing in liquid nitrogen prior to transfer to the precooled bead mill block. Here, the quality of tube used is critical as is the size of ball bearing, which should not surpass 3 mm to minimize the risk of tube rupture during milling and centrifugation.


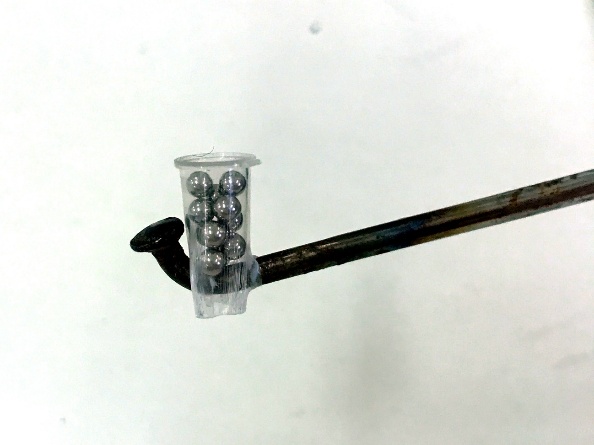


Figure 1: Improvised scoop for measuring a suitable quantity of stainless steel ball bearings for sample maceration.

**Before starting extraction:**

Preheat a water bath or incubator/oven to 65 °C.

Warm the lysis buffer at 65 °C for at least 15 minutes before pipetting/dispensing.

DNA extraction protocol:

1. Add an excess of sorbitol wash buffer to fill sample tubes containing macerated plant material to approximately ¾ capacity (0.9 – 1.5 ml, depending on tubes used). Cap tubes and shake in the bead mill, manually or using vortex. Inspect to confirm suspension of the powdered material and shake again, if necessary.
2. Centrifuge at 2,500 x g for five minutes at room temperature.
3. The supernatant is decanted or aspirated from samples and discarded.
4. (Optional) Repeat the sorbitol wash if the supernatant from step 3 is highly turbid or viscous. Resuspend and centrifuge as before. Discard supernatant. One sorbitol wash cycle is usually sufficient but can be repeated, particularly with samples especially rich in polysaccharide or tannins, judged by the clarity and color of the supernatant after centrifugation.
5. Add pre-warmed extraction buffer to the sample tubes (approximately ½ the sample tube capacity or 500 to 700 µl for example when 1.1 ml microtubes in 96-well are used).
6. Resuspend macerate samples by shaking for five seconds in the bead mill or by vortexing. The ball bearings remaining in the tubes greatly assist the macerate mixing process.
7. Incubate samples at 65°C for a minimum of 30 minutes, up to 60 minutes, with mixing by inversion every ten minutes. Remove from water bath/oven and allow samples to cool at room temperature for five minutes.
8. Add CIA to sample tubes (700 µl or fill to approximately 4/5 tube capacity). Shake tubes vigorously for 10 seconds. This is efficiently accomplished using the bead mill if desired.
9. Centrifuge samples at 2,500 x g for ten minutes at room temperature. (Higher centrifugation speeds were not used, to avoid the risk of tube rupture, especially if larger diameter ball bearings are utilized).
10. Carefully remove the upper aqueous phase by pipetting. Transfer to a new tube, carefully avoiding disturbance of the debris between phases.

Although usually unnecessary, extraction with an equal volume of CIA, could optionally be repeated, with bead mill mixing and centrifugation at 2,500 to 13,000 x g for 10 minutes and recovery of the upper phase to a fresh tube.

When using microtubes in 96-well format, the tube strips should be carefully lifted from the tube rack to allow visual monitoring of this recovery process when using multichannel pipettes.

Ball bearings may be recovered in a sieve and washed thoroughly with tap water. If required, they can be made reusable by soaking in 10 % bleach for ten minutes, washing in four changes of Milli-Q quality H_2_O and drying at 70°C for at least one hour.

1. Nucleic acids are precipitated by the addition of 1/10 volume of 3 M sodium acetate pH 5.2 and 0.66 volumes of cold isopropanol (stored at -20 °C). Mix by inverting ten times and keep at -20 °C for at least one hour or overnight.
2. Centrifuge at 13,000 x g for 10 minutes at room temperature. For microtubes in 96-well format the maximum allowable speed in the plate rotor should be used.
3. Carefully decant off the supernatants and drain tubes by resting inverted on paper towels.
4. Wash pellets by the addition of 1 ml of 70 % ethanol. Centrifuge at 13,000 x g for 10 minutes.
5. Carefully remove supernatants by aspiration to avoid loss of the nucleic acid pellet.
6. Dry open tubes at room temperature for approximately one hour or vacuum dry for 10 minutes.
7. Gently suspend pellets in 100 µl TE containing 0.1 mg ml^-1^ DNase-free RNase A and incubate tubes at 37 °C for 30 minutes.

**DNA extraction notes:**

DNA purity may be conveniently estimated using a spectrophotometer, such as a Nanodrop 2000 (Thermo Scientific). DNA yield is more accurately estimated using a fluorimeter and fluorescent DNA binding dye, such as the Qubit system (Thermo Scientific). Yield may also be verified and DNA integrity checked by agarose gel electrophoresis.

For applications requiring high yields and high molecular weight genomic DNA, such as long read single molecule PacBio sequencing, several grams of fresh tissue should be macerated with a mortar and pestle in the presence of liquid nitrogen. The extraction protocol is scaled up to use 15-ml or 50-ml tubes or several microtubes in parallel and the DNA consolidated at the end. Gentle handling must be adopted throughout the entire procedure with no or only minor vortexing and never any repeated pipetting. Resuspension of the tissue macerate following the sorbitol pre-washes should be carried out gently using a spatula and the final DNA precipitate recovered using a glass hook. DNA integrity can be estimated using pulsed-field gel electrophoresis and the adequate fraction selected for downstream sequencing using the Blue pippin DNA size selection system (BluePippin; Sage Science).

RNA extraction protocol:

For RNA extraction, biological material was kept frozen at all times until the addition of sorbitol buffer. It is possible to freeze tubes containing samples and ball bearings at -80 ^o^C along with the bead mill sample block, before using the bead mill, but samples are frequently found to have thawed by the end of maceration. We also tried freezing tubes in liquid nitrogen before using the bead mill, but frequently we lost samples because of broken tubes. If high throughput is not an issue, it is recommended to macerate tissues using a mortar and pestle in liquid nitrogen for RNA extraction.

Each mortar/pestle and spatula should be previously cleaned using a RNase decontamination solution, rinsed in distilled water, dried and pre-chilled in liquid N_2_ before grinding samples.

**Before starting extraction:**

Preheat a water bath or incubator/oven to 65 °C.

Warm the lysis buffer at 65 °C for at least 15 minutes before pipetting/dispensing.

1. Making sure to keep the tissue frozen with either liquid N_2_ or dry ice, transfer macerated samples into 2.0 ml tubes (if not using the bead mill).
2. Immediately add an excess of sorbitol wash buffer to fill sample tubes containing macerated plant material to approximately ¾ capacity (0.9 – 1.5 ml, depending on tubes used). Cap tubes and shake in the bead mill, using a vortex or manually. Inspect to confirm suspension of the powdered material and shake again, if necessary.
3. Centrifuge at 2,500 x g for five minutes at room temperature.
4. The supernatant is aspirated from samples and discarded.
5. Repeat the sorbitol wash (steps 1 to 3). We routinely used two rounds of the sorbitol solution wash for RNA extraction. Additional washes are unnecessary. RNA extraction is then continued as previously described.
6. Quickly add 1.0 ml of the pre-warmed CTAB extraction buffer, shake vigorously and place in 65 ^o^C water bath. The amount of tissue processed is dependent on type. Approximately 100 µg for leaves, 80 mg for cambium per microtube.
7. Add about 750 µl of chloroform and shake vigorously.
8. Centrifuge for 10 minutes at 12,000 x g.
9. Carefully aspirate the aqueous phase and transfer to a fresh tube.
10. Add 750 µl chloroform. Shake vigorously, centrifuge for 10 minutes at 12,000 x g.
11. Carefully transfer aqueous phase to a fresh tube. Be sure to note the volume recovered.
12. Place tubes on ice and add an equal volume of LiCl_2_:EDTA and mix well.
13. Precipitate for 2 to 4 hours at -20 °C. Alternatively, precipitation can take place overnight at 4°C. However, there is an inverse relationship between quality and yield.
14. Balance pairs of tubes with LiCl_2_:EDTA and centrifuge at 12,000 x g for 20 minutes.
15. Carefully remove and discard the supernatant being sure not to lose the pellet.
16. Dissolve the pellet in 500 or 1000 µl of SSTE (depending on pellet size.) If necessary, heat in a 65 ^o^C water bath for 2 to 10 minutes to help the pellet dissolve. Transfer to a fresh microtube.
17. Extract once with 500 µl chloroform. Shake or vortex tube before centrifuging at full speed for 5 to 7 minutes.
18. Remove aqueous phase to new microtube.
19. Add 2 volumes of 95 % ethanol and precipitate for a minimum of 15 minutes at -80^o^C.
20. Centrifuge for 20 minutes at full speed to pellet the RNA. Pour off and discard supernatant being careful not to lose the pellet.
21. Wash the pellets by adding 200 µl cold 70 % ethanol and centrifuge the samples at full speed for 3 to 5 minutes. Carefully aspirate supernatant as the pellets are very loose after 70 % ethanol wash.
22. Air dry the pellets by leaning the tubes open and on their sides on lab bench, do not speed vac to avoid contamination.
23. After the pellet is dry, add enough sterile MilliQ-grade, RNase free water or TE to dissolve the pellet (usually 10 to 50 µl depending on pellet size).
